# The German version of the self-injurious thoughts and behaviors interview (SITBI-G): a tool to assess non-suicidal self-injury and suicidal behavior disorder

**DOI:** 10.1186/s12888-014-0265-0

**Published:** 2014-09-18

**Authors:** Gloria Fischer, Nina Ameis, Peter Parzer, Paul L Plener, Rebecca Groschwitz, Eva Vonderlin, Michael Kölch, Romuald Brunner, Michael Kaess

**Affiliations:** Section for Disorders of Personality Development, Clinic of Child and Adolescent Psychiatry, Centre of Psychosocial Medicine, University of Heidelberg, Blumenstrasse 8, Heidelberg, 69115 Germany; Department of Child and Adolescent Psychiatry and Psychotherapy, University of Ulm, Ulm, Germany; Institute of Psychology, University of Heidelberg, Heidelberg, Germany; Department of Child and Adolescent Psychiatry, Psychotherapy and Psychosomatic of the Vivantes Netzwerk für Gesundheit GmbH Berlin, Berlin, Germany

**Keywords:** Nonsuicidal self-injury, Suicidal behavior disorder, DSM-5, SITBI-G, Psychometric properties, Adolescents

## Abstract

**Background:**

Self-injurious thoughts and behaviors (SITBs) are common in adolescents. While there is no standardized interview in German to assess SITBs to date, the Self-Injurious Thoughts and Behaviors Interview (SITBI) is widely used in English-speaking countries. However, the SITBI has not been validated for the assessment of the recently issued DSM-5 Section 3 diagnoses of nonsuicidal self-injury (NSSI) and suicidal behavior disorder (SBD) yet. In the present study the psychometric properties of the German version of the SITBI (SITBI-G) were assessed. We also evaluated whether SITBI-G is a reliable and valid instrument to establish diagnoses of NSSI and SBD.

**Methods:**

A clinical adolescent sample (N = 111, f/m = 73/38, age range = 12-19 years) was recruited from the inpatient units of three departments of child and adolescent psychiatry in Germany. All participating patients were interviewed by using the SITBI-G, and DSM-5 criteria of NSSI and SBD were operationalized from the SITBI-G data. Additionally, participants were given the Self-Harm Behavior Questionnaire (SHBQ), and SITBI-G was retested in a subsample.

**Results:**

The SITBI-G shows moderate to good test-retest reliability, a very good interrater reliability, and a good construct validity. The results demonstrate that diagnoses of NSSI and SBD can be established using the SITBI-G, achieving moderate to good test-retest reliabilities and very good to perfect interrater reliabilities.

**Conclusions:**

Overall, the good psychometric properties of SITBI-G are comparable to the original version of the interview. Therefore, SITBI-G seems to be highly appropriate to assess SITBs, including the new DSM-5 Section 3 diagnoses NSSI and SBD in research and clinical contexts.

**Electronic supplementary material:**

The online version of this article (doi:10.1186/s12888-014-0265-0) contains supplementary material, which is available to authorized users.

## Background

Self-injurious thoughts and behaviors (SITBs) are frequently observed in adolescent populations, with up to a third of European students in school populations reporting to have deliberately injured themselves at least once in their lifetime [1]. These behaviors can be distinguished according to the intent to die as “nonsuicidal” or “suicidal” [2]. According to Klonsky and Muehlenkamp, nonsuicidal self-injury (NSSI) is “the intentional destruction of body tissue without suicidal intent and for purposes not socially sanctioned” [3]. In a consecutively recruited sample of adolescent and young adult psychiatric inpatients, 60% of the patients reported engagement in NSSI during the last year, and half (49.6%) even reported repetitive NSSI [4]. Deliberate cutting is the most common form of NSSI, followed by other typical methods such as scratching or biting and also hitting one’s body [5,6]. The arms and wrists in particular are most frequently affected by NSSI [7].

The definition of suicidal behavior usually includes suicidal ideation, suicide plans, suicide attempts, and completed suicide [8]. It is a very common symptom of various psychiatric disorders such as affective disorders and personality disorders [9,10]. Within a clinical sample of adolescents, 74% of the patients reported a history of suicidal thoughts, and 25.6% of them reported to have had at least one suicide attempt in their life [11]. According to the World Health Organization (WHO), suicide is the second leading cause of death in adolescents and young adults [12].

Although nowadays regarded as separate phenomenons, NSSI and suicidal behavior are often associated [13,14]. In many clinical and population-based studies [1,14,15], NSSI has been shown to be a risk factor for suicidal behavior [2,13]. Due to increasing public and scientific interest as well as clinical importance, both NSSI and suicidal behavior have recently been included in section 3 of the new DSM-5 [16,17]. Here, the diagnosis Suicidal Behavior Disorder (SBD) does not include either suicidal ideation or suicidal plans, but only suicide attempts [16].

Four self-assessment tools currently exist in German for assessing SITBs [18]. These include the German version of the Self-Harm Behavior Questionnaire (SHBQ; [19]), the Modified Ottawa/Ulm Self-Injury Inventory (MOUSI; [7]), the Deliberate Self-Harm Inventory (DSHI; [20]), and the Functional Assessment of Self-Mutilation (FASM; [21]). These instruments have already been applied in both school-based and clinical samples [1,4,22]. While there is no standardized interview in German to assess SITBs to date, the Self-Injurious Thoughts and Behaviors Interview (SITBI) is widely used in English-speaking countries [23,24]. The original version of the SITBI has already been proven to be a reliable and valid instrument [23] for assessing a broad range of SITBs, including their frequency, methods, functions, and circumstances. However, the use of the SITBI to assess NSSI and SBD according to DSM-5 criteria has not yet been evaluated.

Hence, the present study aims to assess (1) the psychometric quality of the German translation of the SITBI in its long form; and (2) the validity and reliability of the DSM-5 diagnoses NSSI and SBD assessed by the SITBI-G.

## Methods

### Participants

Clinical subjects were recruited from the inpatient units at the Departments of Child and Adolescent Psychiatry in Heidelberg, Ulm, and Berlin, Germany. The sample was consecutively recruited from May 2012 to January 2013. Adolescents and young adults aged 12-19 years were included. Exclusion criteria were acute psychotic symptoms and acute intent to harm oneself or others which required an immediate intensive psychiatric intervention, as well as impaired intellectual function (IQ < 80) and a lack of knowledge of the German language. All inclusion and exclusion criteria were checked with the attending physician before patients and their caregivers were asked to participate.

A final sample size of N = 111 adolescents and young adults (females: n = 73; 65.8%) participated in the study. Fifteen of them did not complete the Self-Harm Behavior Questionnaire (SHBQ); therefore, the sample for comparing the SITBI-G and the SHBQ was restricted to 96 adolescents. Data from a second interview could be obtained in 36 cases. Analyses of test-retest reliability of the SITBI-G were based upon these numbers. The reason for the high amount of missing data in the second interview was discharge of patients from the hospitals. For the evaluation of interrater reliability, data could be obtained from 22 adolescents. The reason for the relatively small number was that several patients or their parents did not consent to audiotape the interviews.

### Study procedure

The study was approved by the Institutional Review Board (IRB) of the faculty of medicine in Ulm and the IRB of the faculty of medicine in Heidelberg and was carried out in accordance with the Declaration of Helsinki. Participants and their parents/caregivers received a detailed personal and written description of the study and gave their written informed consent, according to the different IRB approvals. All participating patients were interviewed by using the SITBI-G and received the SHBQ. Retests of the SITBI-G were performed in a subsample. The interval between interview 1 and 2 was on average 25 days (Range 5-93 days) dependent on the availability of the patients. If adolescents and caregivers consented, the interviews were audiotaped. Interrater scores were assessed by an associate of the research team in Ulm for interviews conducted in Heidelberg and vice versa. Thus, the selection of ratings was randomized. All interviews were conducted by clinical psychologists or physicians at the masters or M.D. level. The authors extensively trained all interviewers. A monthly peer consulting was arranged to guarantee that the same approach to the SITBI-G was applied.

### Data assessment

Clinical diagnoses according to the ICD-10 diagnostic criteria were established by consensus between two child and adolescent psychiatrists. The first interview assessment comprised a sociobiographic assessment, which included information about gender, date of birth, state of adolescents’ and parents’ education, therapeutic setting (inpatient or daily inpatient), current living situation, and comorbid diagnoses.

The long form of the German version of the SITBI (SITBI-G) was the main assessment tool in the present study. It was translated and back-translated by our scientific working group, which comprised expert members of the Universities of Heidelberg and Ulm. The SITBI was originally developed by Nock and colleagues [23]. The SITBI-G is a semi-structured interview used to assess the presence, frequency, and characteristics of a wide range of SITBs, including suicidal ideation, suicide plans, suicide gestures, suicide attempts, and both thoughts of NSSI and NSSI itself. It contains 169 items in six modules that refer to the six types of SITBs. All modules start with a dichotomized screening question concerning experience of the presence/absence of thoughts or behaviors. In the following, frequencies (lifetime, past year, past month, and past week), functions, triggers, precipitants, and different characteristics (severity, method used, abuse of drugs, peer influence, and future likelihood) are recorded. In order to assess the new diagnoses of NSSI and SBD, the SITBI-G items were matched with the DSM-5 proposed criteria for NSSI, SBD, and current SBD (see Additional file 1: Table A). For some proposed criteria, proper equivalents were found. For others, a workaround was chosen because no proper equivalent was found in the SITBI-G, e.g., Criterion E in NSSI (in the DSM-5 “The behaviour does not occur exclusively during psychotic episodes, delirium, substance intoxication, or substance withdrawal. In individuals with a neurodevelopmental disorder, the behavior is not part of a pattern or repetitive stereotypes. The behavior is not better explained by another disorder or mental condition.”) was verified by the diagnosis of each participant and was only considered to be fulfilled if none of the aforementioned conditions were diagnosed. Administration of the SITBI-G takes between five up to 30 minutes dependent on the occurrence of the different SITBs.

Additionally, the German version of the Self-Harm Behavior Questionnaire (SHBQ) [19,25] was applied in this study. The main reason for using the SHBQ was the contextual overlap with the SITBI-G, which made it possible to examine whether the respective answers in the interview and questionnaire corresponded. The SHBQ consists of 65 items in four modules. Each module starts with a dichotomized screening question. Among others, the frequency, first time and last time, communication, and need for medical treatment with respect to every behavior is recorded. The German version of the SHBQ has been proven to be a reliable instrument to assess SITBs [25].

### Data analyses

Descriptive analyses (M, SD, and frequencies) for the quantitative items of the SITBI-G, the sample description, and the prevalence rates of NSSI, SBD, and current SBD were administered. Cohen’s Kappa (κ) was used to analyze the test-retest reliability, the interrater reliability, and the construct validity of the German version of the SITBI. In accordance with Fleiss, Levin, and Paik a value smaller than .40 can be interpreted as weak agreement, values between .40 and .75 as moderate to good agreement, and values greater than .75 as very good agreement [26]. Cohen’s Kappa was even chosen to be the indicator for agreement between the DSM-5 diagnosis and the results of the SITBI-G. All analyses were performed using Stata 12.

## Results

### Sample characteristics

The mean age of the total sample was 15.38 years (SD = 1.72; range 12-19). In all, 94.5% of the sample was German. 20.2% of the whole sample attended the Hauptschule (nine years of secondary elementary school), 30.3% attended the Realschule (six years of secondary school after four years of elementary school, terminating with a secondary school level-I certificate) and 49.5% attended the Gymnasium (eight years of secondary school after four years of elementary school, terminating with the general qualification for university entrance). Most participants of our sample were treated in an inpatient unit (76.9%; 23.2% were from a day clinic). A wide range of psychiatric comorbidities were identified (for detailed information see Additional file 1: Table B).

The most common diagnoses in the total sample were mood disorders (53.2%), neurotic, stress-related, and somatoform disorders (31.5%), and behavioral and emotional disorders with onset usually occurring in childhood and adolescence (20.7%).

### Descriptive results

Of the diagnoses proposed in the DSM-5 section 3 assessed by the SITBI-G, 31.2% of the patients fulfilled the criteria of SBD and 27.0% of current SBD. Another 36.9% fulfilled those of NSSI. Most common comorbidities of the DSM-5 diagnoses were mood disorders and neurotic, stress-related and somatoform disorders (42.6% and 20.4% for SBD, 42.6% and 21.3% for current SBD and 43.9% and 19.7% for NSSI).

Table 1 presents the frequencies, means, and standard deviations of the presence, frequencies, and functions of the different types of SITBs. The majority of subjects showed at least one type of SITB. Most of the adolescents reported suicidal ideation. The age at onset varied over the different SITBs between 12 and 14 years of age. Severity of the different types of SITBs was consistently reported as moderate to high. The most frequently reported function of SITBs was ‘the possibility to get rid of bad feelings’. The least frequent function was ‘the possibility to get attention’. The only exception from this trend was found in suicide gestures, which were associated with socially positive reinforcement.

**Table 1:** **Frequencies**, **means**, **and standard deviations of the SITBI**-**G** (**n** =**111**)^**a**^

	**Suicidal ideation**		**Suicide plan**		**Suicide gesture**		**Suicide attempt**		**Thoughts of NSSI**		**NSSI**	
	***n***	**%**	***n***	**%**	***n***	**%**	***n***	**%**	***n***	**%**	***n***	**%**
Presence	85	76.6	52	46.9	21	18.9	43	38.7	65	58.6	60	54.6
Past year	74	66.7	47	42.3	14	12.6	30	27.0	59	53.2	51	46.0
Past Month	50	45.1	24	21.6	5	4.5	9	8.1	48	43.2	37	33.3
Past week	25	22.5	8	7.2	0	0.0	2	1.8	30	27.0	23	20.7
	*M*	*SD*	*M*	*SD*	*M*	*SD*	*M*	*SD*	*M*	*SD*	*M*	*SD*
Frequencies												
Lifetime	15.59	33.65	11.65	35.54	2.05	1.32	3.12	2.85	38.95	62.25	102.98	222.98
Past year	7.33	13.61	5.40	14.09	1.38	1.53	1.64	1.95	19.89	33.78	39.60	78.43
Past month	1.92	3.38	0.94	1.71	0.29	0.56	0.24	0.48	3.88	6.06	4.27	5.89
Past week	0.48	0.89	0.19	0.49	0.00	0.00	0.07	0.34	1.06	1.71	0.75	1.28
Age of onset	12.98	2.04	13.69	2.70	14.05	1.75	13.90	2.07	12.77	2.04	12.47	2.31
Severity (worst point)^b^	3.34	0.85	3.44	0.78	-	-	-	-	3.55	0.73	-	-
Severity (average)^b^	2.27	0.85	2.75	0.95	-	-	-	-	2.52	1.05	-	-
Functions^b^												
To get rid of bad feelings	2.69	1.13	2.63	1.30	2.10	1.51	2.70	1.34	3.06	1.22	3.00	1.30
To feel something	1.89	1.59	1.67	1.44	1.00	1.38	1.74	1.54	2.43	1.65	2.46	1.56
To get attention	0.91	1.17	0.62	0.97	2.71	1.68	0.51	0.96	0.80	1.23	0.64	1.13
To get out of doing something	2.35	1.32	2.50	1.39	1.52	1.47	2.28	1.47	1.43	1.38	1.69	1.50

^a^In order to ensure comparability of the results the table was designed in accordance to Nock and colleagues [23]. ^b^Scale of 0 (“low/little”) to 4 (“very much/severe”).

Social triggers and characteristics of SITBs are shown in Table 2. The results imply that the patients’ mental state and problems with their families had a significant influence on the participants’ SITBs. Concomitant use of alcohol or drugs was most often reported in suicide gestures and suicide attempts in comparison to other SITBs.

**Table 2:** **Frequencies**, **means**, **and standard deviations of the SITBI**-**G** (**n** =**111**)^**a**^

	**Suicidal ideation**		**Suicide plan**		**Suicide gesture**		**Suicide attempt**		**Thoughts of NSSI**		**NSSI**	
	***M***	***SD***	***M***	***SD***	***M***	***SD***	***M***	***SD***	***M***	***SD***	***M***	***SD***
Precipitants^b^												
Family	2.14	1.43	2.08	1.44	1.90	1.58	2.09	1.44	2.20	1.44	2.19	1.48
Friends	1.18	1.34	1.35	1.41	1.38	1.86	1.19	1.45	1.40	1.46	1.47	1.49
Relationships	1.11	1.38	1.31	1.50	1.48	1.60	1.09	1.32	1.26	1.50	1.22	1.51
Peers	1.39	1.36	1.13	1.33	1.10	1.58	1.00	1.36	1.12	1.31	1.20	1.39
Work/school	1.68	1.45	1.98	1.32	0.90	1.41	1.47	1.37	1.62	1.54	1.58	1.52
Mental state	3.14	1.06	3.35	0.88	2.38	1.24	3.33	0.97	3.22	1.01	3.15	1.20
Characteristics												
Pain^b^	-	-	-	-	-	-	1.70	1.50	-	-	-	-
Alcohol/drugs (% of time)	14.64	28.05	15.33	28.92	21.62	37.75	22.56	36.72	11.74	22.40	8.22	17.82
Number of peers with behavior before onset	0.75	1.72	0.60	1.55	5.76	21.49	3.26	15.13	1.95	3.59	1.97	3.77
Number of peers with behavior after onset	2.80	10.85	2.88	13.72	5.43	21.50	3.67	15.07	5.65	13.54	4.62	7.00
Peer influence before onset^b^	0.41	1.00	0.16	0.69	0.88	1.13	0.42	1.12	0.95	1.36	0.80	1.29
Peer influence after onset^b^	0.88	1.23	0.48	1.08	0.50	1.07	0.50	1.06	0.81	1.12	0.81	1.21
Future likelihood of behavior^b^	2.00	1.41	1.75	1.25	1.00	1.05	1.60	1.29	2.45	1.51	2.12	1.58

^a^In order to ensure comparability of the results the table was designed in accordance to Nock and colleagues [23]. ^b^Scale of 0 (“low/little”) to 4 (“very much/severe”).

Adolescents reported having fewer friends engaging in the same behaviors before their act than afterwards, with the exception of suicide gestures. In addition patients reported that having friends with SITBs did neither affect the patient’s SITBs onset nor the maintenance. Regarding the probability of future SITBs, the reported probabilities were highest in thoughts of NSSI and NSSI.

### Test-Retest Reliability

The examination of test-retest reliability of the SITBI-G was based on comparing the data of the SITBI-G at the first interview assessment with those at follow-up. Regarding the test-retest reliability for the DSM-5 diagnosis, results reveal moderate to good agreements for SBD (κ = .64), current SBD (κ = .52), and NSSI (κ = .60).

The results for all different SITBs are presented in Table 3 and show good to very good agreements for the presence of all SITBs. With exception of the bad agreement in the test-retest reliability for the lifetime frequency of suicide gestures and suicide attempts, moderate to very good agreements were found for the lifetime frequency of suicidal thoughts, suicidal plans, thoughts of NSSI, and NSSI. The results for the frequency of SITBs in the past year range from good to very good. The only exception constituted the weak agreement for suicide gestures. The test-retest reliability of the functions varied over the different types of SITBs from predominantly weak to even very good agreements.

**Table 3:** **Results of the analysis of the Test**-**Retest Reliability of the SITBI**-**G** (**n** = **36**)

	**Suicidal ideation**	**Suicide plan**	**Suicide gesture**	**Suicide attempt**	**Thoughts of NSSI**	**NSSI**
	**κ**	**κ**	**κ**	**κ**	**κ**	**κ**
Presence	.72	.83	.75	.75	.88	.70
Frequency (lifetime)	.85	.43	-.11	.18	.67	.48
Frequency (past year)	.79	.53	-.07	.50	.81	.64
Functions						
To get rid of bad feelings	.06	.46	.30	.22	.32	.34
To feel something	.34	.22	.68	.33	.34	.27
To get attention	.45	.29	.29	.89	.50	.49
To get out of doing something	.30	.28	.25	.47	.28	.46

### Interrater reliability

The examination of the interrater reliability of the SITBI-G was based on the data from all those interviews for which the patients had consented to record the interview (n = 22). Regarding the interrater reliability for the DSM-5-diagnoses calculated on the basis of the agreement between the data of the SITBI-G and the rater’s data, the results revealed very good agreement for NSSI (κ = .77) and perfect agreement for SBD (κ = 1.00) and current SBD (κ = 1.00). Results for the lifetime prevalence of each SITB implied consistently perfect agreements (all κs = 1.00). Continuously excellent agreements were also found for the frequency of suicidal ideations, suicide gestures, suicide attempts, and thoughts of NSSI over lifetime (all κs = 1.00). A very good agreement was found for lifetime frequency of NSSI (κ = .80). Excellent interrater reliabilities were present for the frequency of suicidal ideations, suicide plans, suicide attempts, and NSSI in the past year (all κs = 1.00). Very good interrater reliabilities were reached for the frequency of suicide gestures (κ = .81) and thoughts of NSSI (κ = .90) in the past year. Similar results were found regarding the examination of the functions for all types of SITBs (all κs = .77-1.00).

### Construct validity

To examine the construct validity of the SITBI-G, the data from the interview were compared with the data from the SHBQ insofar as a match of content was given. Results showed very good agreements for the presence of NSSI (κ = .89) and suicide attempts (κ = .86). Moreover, moderate to good agreements were found for the presence of suicide plans (κ = .58) and suicidal ideation (κ = .75). In contrast, weak agreement was again found for the presence of suicide gestures (κ = .37). Referring to the frequency of SITBs over lifetime, the results implied no agreement between the SITBI-G and the SHBQ for suicide gestures (κ = -.28) but also a moderate agreement for suicide attempts (κ = .45).

## Discussion

### Characteristics of SITBs

In the present study, prevalence rates of SITBs are in line with the current literature [4,27,28]. Of the participants, 31.2% met the diagnostic criteria for a diagnosis of SBD and 27.0% of current SBD; 36.9% met the criteria for a diagnosis of NSSI. These numbers are comparable to previously reported prevalence rates for NSSI and suicidal behavior in adolescents [4,27,28]. With regards to the DSM-5 diagnosis of NSSI, Barrocas and colleagues recently reported a rate of 1.5% in a sample of school children (n = 665) interviewed with the SITBI [24]. Our study underlines the high prevalence of NSSI in clinical samples of adolescents when using the same assessment instrument. The main age at onset of SITBs varied between 12 and 14 years. These ages at onset are often found in the literature published on NSSI [2,28-31] and all types of SITBs [23,32].

The four functions of NSSI assessed in the SITBI correspond to the common descriptions reported by Nock & Prinstein [27]: “getting rid of bad feelings” corresponds to automatic negative reinforcement; “to feel something” corresponds to automatic positive reinforcement; “to get attention” corresponds to social positive reinforcement and “to get out of doing something” corresponds to social negative reinforcement. Most of the adolescents reported that they engaged in SITBs to get rid of bad feelings regardless of their suicidal intent. This result is concordant with the comprehension of NSSI as a coping strategy for dealing with aversive emotional states [33]. Notably, suicide gestures differed from this pattern: Patients reporting suicide gestures also reported getting attention as the most important function of their behavior. In comparison with the other SITBs, the social impact was higher in suicide gestures. In combination with our finding of being aware of more friends with a suicide gesture before the act, this presents an interesting picture of this construct and underlines its separate nature, as proposed by the model of Nock [2].

### Psychometric properties

The results of the test-retest reliability of the SITBI-G are mainly congruent with the findings of the original version of the SITBI in English [23]. However, the results for suicide attempts and NSSI differ from the findings of Nock [23]. A reason for this result could be related to the psychological impairment in the clinical sample: Considering the increasing frequency of NSSI between interviews at time 1 and time 2, participants’ NSSI may have been triggered by other patients’ NSSI during their inpatient treatment or, alternatively, treatment may have increased participants’ willingness to report SITBs.

Results regarding the interrater reliability of the SITBI-G are in line with the findings by Nock and colleagues [23]. Thus, very good to perfect agreements were found for the presence and the frequency of the different types of SITBs over lifetime. Existing differences can be explained to some extent by nonspecific answers of the patients and the absence of a clear definition for suicide attempts.

For the analysis of the construct validity of the SITBI-G, the SHBQ was consulted as a comparative diagnostic instrument in the present study, whereas Nock and colleagues [23] used other self-assessment tools. Nevertheless, parallels can be found. Thus, similar to the study in the original version of the SITBI, good to very good agreements were found for the presence of NSSI, suicide attempts, and suicidal ideation. Concurrently, the results of the present study implied bad to weak agreements for the presence of suicide gestures and suicide attempts over lifetime. This could be due to different definitions of those behaviors in the instruments. Moreover, we experienced that patients had problems understanding the concept of suicide gestures in the interview, which was then explained by the interviewer. This problem may also have occurred when completing the questionnaire.

The weak reliability of suicide gestures was an overall problem in both the present and the original study. Nock and colleagues mentioned the lack of clarity in the concept of suicide gestures, the less severe consequences of suicide gestures in comparison to the consequences of other SITBs being given to explain why the probability was higher to forget over time [23]. Moreover, suicide gestures comparatively fulfill a more social function than the other SITBs [34]. These functions seem to be more detached from the patients than the more self-involving functions, e.g., to get rid of bad feelings, which may lead to a less valid answer.

The reliability of the NSSI and SBD diagnosis, as suggested in the DSM-5, assessed by the SITBI-G, was investigated. Although the SITBI was originally not designed to assess the DSM-5 criteria, the items fit the DSM-5 criteria very well. Moreover, the good to perfect interrater and test-retest reliabilities underline the goodness of the SITBI-G as a useful tool to assess the new criteria for NSSI and SBD. In general, the authors agree with Barrocas and colleagues that the SITBI presents a feasible option to estimate DSM-5 defined diagnoses of both NSSI and SBD [24].

With regard to future studies, further development of the SITBI-G would be desirable to render the interview even more comprehensible and economical. In this context a detailed definition of suicide gestures would strengthen the validity of the patients’ answers. Additionally, we should consider revising the answer scale in order to have the possibility to answer “no” instead of “low/little” if the answer to an item does not apply. Moreover, the testing of a possible correlation between SITBs and specific psychiatric diagnoses would be interesting, whereby a standardized diagnostic procedure would be desirable for the objectification of the diagnoses. With regards to the newly proposed diagnostic criteria for SBD and NSSI in the DSM-5 and the possibility to use the SITBI as a clinical interview to assess these criteria, the SITBI should be modified. For example, items to assess SBD in the last two years and items to assess the state of awareness during the suicide attempt should be added, as well as asking whether the attempt was undertaken in a religious or political context. In NSSI, items concerning the functions of SITBs (e.g., to induce positive feelings) and items regarding the reasons for SITBs (e.g., depression, anxiety, and tension) should be extended and it should also be asked whether the behavior or its consequences causes clinically significant distress, for example.

The findings of the present study are subject to some limitations. First, no objective data were used (i.e., medical estimates) for patients’ self-injurious behaviors to validate the data obtained by the SITBI-G. In reference to that, Plener and colleagues point to the fact that some patients exaggerate their statements on the severity and the extent of their self-injuries, which can also have an impact on the data of the interview [22]. Additionally, by using the SITBI-G to assess the DSM-5 diagnosis of SBD and NSSI we could not assess every criterion mentioned in the DSM-5. For example, the E criterion (functional impairment) in NSSI was not specially assessed through the SITBI-G; however, given that the study sample comprised psychiatric inpatients, the authors assumed it to be fulfilled. Another difficulty of the SITBI-G was found with regards to the time frame of the SBD diagnoses. Current SBD was assessed based on the question “How many suicide attempts have you made in the past year?”, whereas the two-year timeframe of the SBD diagnosis had to be calculated based on the question “When was the most recent attempt?”, which in some cases produced results that were not concrete because of the participants’ memory bias.

A particular strength of the present study is the reasonably large clinical sample of adolescents from different clinics, which is typical for German child and adolescent psychiatry. In particular, we can highlight the fact that this is the first study worldwide to assess the DSM-5 diagnosis of SBD and NSSI with the SITBI-G in a clinical sample.

## Conclusions

Overall, the German version of the SITBI has comparatively good psychometric qualities that are predominantly in line with the original English version. Therefore, the SITBI-G can be considered a suitable instrument to assess various types of SITBs. Concurrently, it is a useful and reliable tool to assess the new DSM-5 criteria for NSSI and SBD. As it has been pointed out by Groschwitz et al. [18] the SITBI-G can be used as an in-depth assessment of both NSSI and suicidal behavior. The SITBI-G allows evaluating both phenomenology and severity of SITBs as well as the differentiation of these phenomena. Conducting the interview could be used as introduction to a discussion about the themes of NSSI and suicidality in psychotherapy. A detailed assessment of the underlying motives/functions of SITBs appears also valuable for the selection of specific therapeutic strategies. In addition, the SITBI-G can be well applied in research, i.e., to measure changes in treatment studies. However, the use of the SITBI-G in epidemiological studies is limited, due to the length of assessment.

## Additional file

Additional file 1: Table A. Operationalization of the diagnoses suicidal behavior disorder and nonsuicidal self-injury proposed in the DSM-5 by using the SITBI-G. **Table B.** Diagnostic categories for the whole sample and both groups (Heidelberg and Ulm/Berlin), respectively.

## Abbreviations

DSHI: Deliberate Self-harm Inventory
FASM: the Functional Assessment of Self-Mutilation
IRB: Institutional review boards
MOUSI: Modified Ottawa/Ulm Self-Injury Inventory
NSSI: Nonsuicidal self-injury
SBD: Suicidal behavior disorder
SHBQ: Self-Harm Behavior Questionnaire
SITBs: Self-injurious thoughts and behaviors
SITBI: Self-Injurious Thoughts and Behaviors Interview
SITBI-G: Self-Injurious Thoughts and Behaviors Interview German
WHO: World Health Organization
