# Supplementary material for: The German version of the self-injurious thoughts and behaviors interview (SITBI-G): a tool to assess non-suicidal self-injury and suicidal behavior disorder
Source: BMC Psychiatry. 2014 Sep 18;14:265. doi: 10.1186/s12888-014-0265-0 (PMC4174267; doi:10.1186/s12888-014-0265-0)
Supplement: Additional file 1: Table A. — Operationalization of the diagnoses suicidal behavior disorder and nonsuicidal self-injury proposed in the DSM-5 by using the SITBI-G. Table B. Diagnostic categories for the whole sample and both groups (Heidelberg and Ulm/Berlin), respectively. [file 12888_2014_265_MOESM1_ESM.docx]

**Table A**

Operationalization of the diagnoses suicidal behavior disorder and nonsuicidal self-injury proposed in the DSM-5 by using the SITBI-G

| DSM 5 criteria | Questions in the SITBI-G |
| --- | --- |
| **Suicidal behavior disorder** |  |
| A Within the last 24 months, the individual has made a suicide attempt.  *Current*: Not more than 12 months since the last attempt. | 86) When was the most recent attempt?  89) How many have you made in the past year? |
| B The act does not meet criteria for nonsuicidal self-injury, that is, it does not involve self-injury directed to the surface of the body undertaken to induce relief from negative feeling / cognitive state or to achieve a positive mood state. | Yes in the screening Question suicidal intent. |
| C The diagnosis is not applied to suicidal ideation or to preparatory acts. | Yes in the screening Question suicidal intent |
| D The act was not initiated during a state of delirium or confusion. | Yes in the screening Question suicidal intent |
| E The act was not undertaken solely for a political or religious objective. | 93) What were the circumstances that contributed most to your most recent attempt?  98) Why do you think you make suicide attempts? |
| **Nonsuicidal self-injury** |  |
| A In the last year, the individual has, on 5 or more days, engaged in intentional self-inflicted damage to the surface of his or her body of a sort likely to induce bleeding, bruising, or pain (e.g., cutting, burning, stabbing, hitting, excessive rubbing), with the expectation that the injury will lead to only minor or moderate physical harm (i.e., there is no suicidal intent). | 120) How many separate times in the past year? |
| B The individual engages in the self-injurious behavior with one or more of the following expectations:  1. To obtain relief from a negative feeling or cognitive state.  2. To resolve an interpersonal difficulty.  3. To induce a positive feeling state. | 126) On a scale of 0 to 4, how much did you think of engaging in NSSI as a way to get rid of bad feelings?  130) On a scale of 0 to 4, to what extent did problems with your family lead to you having thoughts of engaging in NSSI?  131) How much did problems with your friends lead to these thoughts?  132) How much did problems with your relationships lead to these thoughts?  133) How much did problems with your peers lead to these thoughts? |
| C. the intentional self-injury is associated with at least one of the following:  1.Interpersonal difficulties or negative feelings or thoughts, such as depression, anxiety, tension, anger, generalized distress, or self-criticism, occurring in the period immediately prior to the self-injurious act  2. Prior to engaging in the act, a period of preoccupation with the intended behavior that is difficult to control.  3. Thinking about self-injury that occurs frequently, even when it is not acted upon. | 125) Why do you think you have thoughts of engaging in NSSI?  116) Have you ever had thoughts of purposely hurting yourself without wanting to die? |
| D. the behavior is not socially sanctioned (e.g., body piercing, part of a religion or cultural ritual) and is not restricted to picking a scab or nail biting. | 150) Now I’m going to go through a list of things that people have done to harm themselves. Please let me know which of these you’ve done: 1) cut or carved skin  2) hit yourself on purpose  3) pulled your hair out 4) gave yourself a tattoo  5) picked at a wound  6) burned your skin (i.e., with a cigarette, match or other hot object) 7) inserted objects under your nails or skin 8) bit yourself (e.g., your mouth or lip) 9) picked areas of your body to the point of drawing blood 10) scraped your skin 11) “erased” your skin to the point of drawing blood 12) other (specify):___________________________ 88) not applicable 99) unknown |
| E. The behavior or its consequences cause clinically significant distress or interferences in interpersonal, academic, or other important areas of functioning. | Criterion E was considered to be fulfilled due to the fact that the study population was a clinical inpatient sample. |
| F. The behavior does not occur exclusively during psychotic episodes, delirium, substance intoxication, or substance withdrawal. In individuals with a neurodevelopmental disorder, the behavior is not part of a pattern or repetitive stereotypes. The behavior Is not better explained by anther mental disorder or medical condition (e.g., psychotic disorder, autism spectrum disorder, intellectual disability, Lesch-Nyhan syndrome, stereotypic movement with self-injury, trichotillomania [hair pulling disorder], excoriation [skin-picking] disorder). | Criterion F was considered to be fulfilled because of the diagnoses distribution of the study population. |

**Table B**

*Diagnostic categories for the whole sample and both groups (Heidelberg and Ulm/Berlin), respectively.*

| Diagnoses (ICD-10)^a^ | | N | % |
| --- | --- | --- | --- |
|  | F1 (Mental and behavioral disorders due to psychoactive substance use) | 6 | 5.4 |
|  | F2 (Schizophrenia, schizotypic, and delusional disorders) | 8 | 7.2 |
|  | F3 (Mood [affective] disorders) | 59 | 53.2 |
|  | F4 (Neurotic, stress-related, and somatoform disorders) | 35 | 31.5 |
|  | F5 (Behavioral syndromes associated with physiological disturbances and physical factors) | 20 | 18.0 |
|  | F6 (Disorders of adult personality and behavior) | 12 | 10.8 |
|  | F8 (Disorders of psychological development) | 9 | 8.1 |
|  | F9 (Behavioral and emotional disorders with onset usually occurring in childhood and adolescence) | 23 | 20.7 |

*Note.* ^a^  Multiple diagnoses per participant possible
